# Deubiquitinating enzymes USP4 and USP17 finetune the trafficking of PDGFRβ and affect PDGF-BB-induced STAT3 signalling

**DOI:** 10.1007/s00018-022-04128-1

**Published:** 2022-01-21

**Authors:** Niki Sarri, Kehuan Wang, Maria Tsioumpekou, Casimiro Castillejo-López, Johan Lennartsson, Carl-Henrik Heldin, Natalia Papadopoulos

**Affiliations:** 1 Department of Medical Biochemistry and Microbiology, Uppsala University, Box 582, 75123 Uppsala, Sweden; 2 Department of Pharmaceutical Biosciences, Uppsala University, Uppsala, Sweden; 3 Department of Immunology, Genetics and Pathology, Uppsala University, Uppsala, Sweden

**Keywords:** PDGFRβ, Ubiquitination, STAT3, Receptor tyrosine kinase

## Abstract

Interaction of platelet-derived growth factor (PDGF) isoforms with their receptors results in activation and internalization of receptors, with a concomitant activation of downstream signalling pathways. Ubiquitination of PDGFRs serves as a mark to direct the internalization and sorting of the receptors. By overexpressing a panel of deubiquitinating enzymes (DUBs), we found that USP17 and USP4 efficiently deubiquitinate PDGF receptor β (PDGFRβ) and are able to remove both Lys63 and Lys48-linked polyubiquitin chains from the receptor. Deubiquitination of PDGFRβ did not affect its stability, but regulated the timing of its trafficking, whereby USP17 prolonged the presence of the receptor at the cell surface, while USP4 affected the speed of trafficking towards early endosomes. Induction of each of the DUBs in BJhTERT fibroblasts and U2OS osteosarcoma cells led to prolonged and/or shifted activation of STAT3 in response to PDGF-BB stimulation, which in turn led to increased transcriptional activity of STAT3. Induction of USP17 promoted acute upregulation of the mRNA expression of STAT3-inducible genes* STAT3*,* CSF1*,* junB* and* c-myc*, while causing long-term changes in the expression of* myc* and *CDKN1A*. Deletion of USP17 was lethal to fibroblasts, while deletion of USP4 led to a decreased proliferative response to stimulation by PDGF-BB. Thus, USP17- and USP4-mediated changes in ubiquitination of PDFGRβ lead to dysregulated signalling and transcription downstream of STAT3, resulting in defects in the control of cell proliferation.

**Supplementary Information:**

The online version contains supplementary material available at 10.1007/s00018-022-04128-1.

## Introduction

The platelet-derived growth factor (PDGF) isoforms are mitogens and chemotactic agents for smooth muscle cells, pericytes, fibroblasts and glial cells of the brain [23], regulating embryonic development, would healing, interstitial fluid pressure and blood–brain barrier [3]. PDGF isoforms bind to receptor tyrosine kinases (RTKs) α and β (PDGFRα and PDGFRβ, respectively), causing receptor dimerization and autophosphorylation. This results in internalization and activation of downstream signalling pathways [22, 30] at the plasma membrane as well as during endosomal trafficking [39, 40, 53] and via translocation to the nucleus [41].

Ubiquitination is a post-translational modification of cellular proteins that plays important roles in, e.g., proteasomal degradation of proteins, intracellular trafficking, receptor internalization and downregulation, DNA repair and cell proliferation [43]. Ubiquitination of RTKs following ligand binding and phosphorylation is a hallmark of receptor-mediated endocytosis and serves to mediate interactions of RTKs with the sorting machineries at the cell surface and at endosomes [15]. The importance of addition of ubiquitin moieties for the function of PDGFRβ has been extensively investigated [31, 44, 46], while no deubiquitinating enzyme (DUB) that may regulate the levels of ubiquitination of PDGFRβ has been identified to date.

DUBs have been implicated in the regulation of diverse cellular processes, such as cell cycle, signal transduction, apoptosis, DNA repair and chromatin remodelling [45]. The DUB family consists of approximately 120 members, which can cleave polyubiquitin chains or remove the ubiquitin moiety from target proteins, thus reversing the effect of ubiquitin ligases [29]. DUBs are categorized into several subclasses, the largest being the group of ubiquitin-specific cysteine proteases (USPs) that contain a core USP catalytic domain, a zink-binding motif and a conserved cysteine residue in the catalytic triad [55]. Roles in receptor endocytosis and trafficking have been described for several DUBs [36]. For example, USP8 regulates early-to-recycling endosomal circuit of EGFR subcellular trafficking [2], while AMSH aids the sorting of EGFR to the lysosomal degradation pathway by interacting with the ESCRT machinery [37]. In addition, USP17 was found to be required for clathrin-mediated endocytosis of EGFR and was proposed to have a general role in endocytosis [26] and also to affect cell migration and plasma membrane localization of H-Ras by acting on isoform 2 of Ras converting enzyme 1 (RCE1) at the endoplasmic reticulum [9, 10, 27, 38].

DUBs display strong regulatory connection not only to intracellular sorting routes, but also to various secondary messengers of activated signalling pathways. USP4 is an oncoprotein [17, 18] which is phosphorylated by Akt; it shuttles between the cytoplasm and the nucleus [49] and regulates cell growth pathways by acting on signalling molecules, such as PDK1 and mTOR [11]. Signalling by signal transducer and activators of transcription proteins (STATs) are examples of pathways which are activated downstream of PDGFRβ directly by its intrinsic kinase activity [52, 54] in pre-assembled complexes by the cell membrane [42, 47] or indirectly by the nonreceptor tyrosine kinase Src that is activated by PDGFRs [54]. Notably, PDGF-induced Src-mediated signalling by STAT3 was found to be one of the crucial pathways required for cellular transformation [51], but the mechanism of activation of STAT3 remains enigmatic. Depending on the strength and the nature of the signal, STAT3 can be activated at the plasma membrane, at the perinuclear endosomal compartment as was shown for c-Met [28], in the Golgi [8] or in the nucleus itself [32]. Importantly, it has been demonstrated that PDGFRβ internalization in response to PDGF is a necessary pre-requisite for the full activation of STAT3 [25].

In this work, we show that the ubiquitin-specific proteases USP17 (also known as DUB3) and USP4 efficiently remove ubiquitination of PDGFRβ, affecting its internalization and early-endosomal sorting, thereby controlling the timing of PDGFRβ-induced STAT3 activation, which in turn affects transcription of STAT3-inducible genes, including *STAT3, CSF1, junB, c-myc* and *CDKN1A*, adversely affecting cell proliferation.

## Materials and methods

### Reagents and antibodies

Primary antibodies against Flag-M2 (#F1804) and α-tubulin (#T6074) were purchased from Sigma, and antibodies against HA-tag (sc-805) and ubiquitin (#16-6078-82) from Santa Cruz Biotechnology and Invitrogen, respectively. Polyclonal antibodies recognizing PDGFRβ (CTβ) and Alix (HP95) were homemade [19, 31], and a PDGFRβ antibody was purchased from Biotechne (#AF385). Primary antibodies against phosphorylated PDGFR (pTyr857, #3170), Akt (#9272S), phosphorylated Akt (pSer473, D9E, #4060 / pThr308, 244F9, #4056), p44/p42 MAPK (Erk1/2, 137F5, #4695), phosphorylated p44/p42 MAPK (pErk1/2, pThr202/pThr204, #9101), PLCγ1 (#2822), phosphorylated PLCγ1 (pTyr783, #2821), STAT3 (79D7, #4904) and phosphorylated STAT3 (pTyr705, D3A7, #9145) were purchased from Cell Signalling Technology. A USP4 antibody was from Bethyl Labs (#A300-829A) and a USP17L24 antibody from Abgent (#AP5491b). Secondary antibodies for immunoblotting, HRP-conjugated goat antimouse IgG (#62–6520) goat antirabbit IgG (#65–6120), were from Invitrogen. Puromycin was purchased from Invivogen and doxycycline from TakaraBio.

### Cell culture and treatments

The human embryonic kidney cell line HEK293T, human osteosarcoma cell line U2OS (Uppsala University) and human foreskin fibroblast cell line BJhTERT (Clontech) were cultured in Dulbecco’s Modified Eagle’s medium (DMEM) (Sigma-Aldrich), supplemented with 10% fetal bovine serum (FBS) (Biowest) at 37 °C in 5% CO_2_ humidified atmosphere. The BJhTERT doxycycline-inducible cell lines were cultured in DMEM, supplemented with 0.8 μg/ml puromycin and 10% FBS, while the media for doxycycline-inducible U2OS cells was supplemented with 2.0 μg/ml puromycin and 10% FBS. Starvation media was DMEM, supplemented with 0.1% FBS. Cell monolayers were stimulated with 20 ng/ml PDGF-BB (Chiron Corp.) for biochemical experiments and with a range of concentration from 1 to 20 ng/ml for functional experiments. For stability experiments, cells were pretreated with 50 µg/ml cycloheximide for 1 h. For the induction of USP4 or USP17L22, tet-inducible BhTERT and tet-inducible U2OS cell lines were pretreated with 100 ng/ml doxycycline for 48 h.

### Generation of tet-inducible cell lines

Lenti-X Tet-One Inducible Expression System (Takara Bio USA) was used for the generation of tet-inducible cell lines. Flag-USP4 and Flag-USP17L22 were cloned into the pLVX-TetOne vector, using In-Fusion HD Cloning Kit (Takara Bio USA) and the constructs were tested by transient transfection. Nanoparticle complexes were produced from the Lenti-X vectors using Lenti-X Packaging Single Shots (VSV-G) and transfected to the 293 T cells cultured in 10-cm dishes in 8 ml DMEM supplemented with 10% FBS to obtain enough lentivirus. After 24 h, 8 ml of media was collected and stored at 4 °C; 5 ml of fresh media was added and collected after another 24 h. The collected supernatants were diluted thrice with the media and added to BJhTERT cells or U2OS cells cultured in 6-well plates supplemented with 8 μg/ml polybrene. The media was changed after 16 h to DMEM supplemented with Tet System Approved FBS (Takara Bio USA) and puromycin (0.8 μg/ml for BJhTERT cells, 2.0 μg/ml for U2OS cells). Surviving cells were cultured in media with puromycin. Doxycycline (100 ng/ml) was added to induce the expression of USP4 or USP17L22.

### Preparation of a DUB cDNA library

A cDNA library consisting of 64 Flag- and HA-tagged deubiquitinating enzymes (DUBs) in retroviral expression vectors (backbone vector MSCV-N-Flag-HA-IRES-PURO) [50] was kindly provided by Dr Peter ten Dijke, Leiden, Netherlands. Heat-shock transformation of *E. coli* competent cells with the respective deubiquitinase constructs was performed and the transformed cells were then spread on LB plates (containing 100 μg/ml ampicillin) and incubated at 37 °C overnight. A single colony was then selected, inoculated in 100 ml LB medium, supplemented with 100 μg/ml ampicillin and incubated overnight in a shaker at 37 °C. DNA purification was subsequently performed following the Plasmid DNA purification Nucleobond® Xtra protocol (Macherey–Nagel) and DNA constructs were stored at − 20 °C until further use.

###  Plasmid and siRNA transfection

HEK293T cells were transiently transfected with Lipofectamin 2000 reagent (Invitrogen, USA), and U2OS cells were transfected with Lipofectamine 3000 (Invitrogen, USA), according to the manufacturer’s instructions. For siRNA knockdown, cells were transiently transfected with 10 nM of trilencer-27 USP4 siRNA (#SR305038, sequence A or C, OriGene, USA) or 60 nM of trilenser-27 USP17L9P siRNA (#SR318236, sequences A and C, Origene, USA). Transfection of siRNA was mediated by SilentFect (BioRad Laboratories AB) for 72 and 96 h. The knockdown efficiency was determined by immunoblotting.

### Mutation of the USP4 gene using CRISPR-Cas9 lentiviral transduction

Single guide RNAs (sgRNA) were designed using the online tool at www.broadinstitute.org/gpp/public/analysis-tools/sgrna-design, and cloned into the BsmBI site of the lentiCRISPRv2 lentiviral vector according to [48]. Two sgRNA were designed to target two distinct sequences within exon 4 of the human *USP4* gene according to the GTEx Portal (www.gtexportal.org). Exon 4 is the first constitutive exon, not subjected to alternative splicing, and codes for a conserved domain of the ubiquitin carboxyl-terminal hydrolase 4 protein family. The sgRNA sequences were: G67, 5ʹ-CTATGTATTGGTCCCTACCGagg-3ʹ and G57, 5-cccTACCGAGGCGTGGAATAAAC-3ʹ (PAM sequences shown in lower case letters). Lentiviruses containing the sgRNA, the Cas9 nuclease and puromycin N-acetyl-transferase genes were generated in HEK293T cells by co-transfection of the packaging plasmids psPAX.2 and psMD2 (Addgene). Supernatants containing lentivirus were harvested 24 h and 48 h post-transfection. Lentivirus expressing EGFP based on the pLJM1-EGFP plasmid was used as controls of transduction. The cloned guide RNAs were verified by Sanger sequencing. The subsequent transduction of the U2OS and BJhTERT cells was carried out overnight in OptiMEM (Gibco) containing 8 µg/ml hexadimethrine bromide (polybrene; Sigma-Aldrich) and selection of the transduced cells was performed for 3 days with 2 µg/ml puromycin (Gibco) for U2OS and 0.8 µg/ml puromycin for BJhTERT. Confirmation of successful CRISPR-Cas9 genome editing for USP4 was also determined at the RNA level by qRT-PCR, and at the protein level by immunoblotting. Single clones were isolated by serial dilution in 96-well plates and verified as above.

### Immunoprecipitation and immunoblotting

After starvation and stimulation of 50% confluent cell monolayers with PDGF-BB (20 ng/ml) for the indicated time periods, cells were washed once in ice-cold phosphate-buffered saline (PBS) and lysed in RIPA lysis buffer (0.5% deoxycholate, 0.1% SDS, 1% Triton X-100, 10% glycerol, 20 mM Tris, pH 7.4, 150 mM NaCl), supplemented with 1 mM Pefa Block and 1 mM sodium orthovanadate for 15 min on ice. For co-immunoprecipitation, starved cells were lysed in a mild buffer (1% Triton X-100, 20 mM Tris, 150 mM NaCl, pH 7.5) supplemented with 1 mM Pefabloc and 1 mM sodium orthovanadate. The cell lysates were precleared by centrifugation at 13,000 rpm for 15 min at 4 °C and were incubated with the primary antibody overnight at 4 °C with end-over-end rotation, followed by an one-hour incubation with protein A/G magnetic beads (Pierce, ThermoScientific). The beads were washed three times with the ice-cold lysis buffer and the adsorbed proteins were eluted in 1% of sodium dodecylsulfate (SDS) sample buffer by boiling at 95 °C for 5 min. The protein samples were subjected into SDS–polyacrylamide gel electrophoresis (PAGE); after electro-transfer to PVDF membranes (Immobilon), the membranes were blocked in 5% bovine serum albumin (BSA) in PBS, 0.1% Tween-20 and incubated at 4 °C overnight with primary antibodies, which were prepared according to the suppliers’ recommendations. After three washes in PBS, 0.1% Tween-20, the membranes were incubated with horseradish peroxidase-conjugated secondary antibodies for one hour at room temperature. The proteins were visualized with the enhanced chemiluminescence (ECL) detection system on a charge-coupled device (CCD) camera (BioRad) and quantified using Bio-Rad ImageLab 6.0.1 software. Whenever fluorescent immunoblotting was used, membranes were blocked with Intercept blocking buffer (#927-60001 LI-COR Biosciences, diluted 1:3 in TBS) for 2 h at room temperature, incubated overnight with primary antibodies at 4 °C, washed three times in 0.05% Tween-20 in TBS for 15 min and incubated with fluorescent conjugated secondary antibodies (Alexa680 and IRDye800) diluted in blocking buffer, followed by three washes in 0.05% Tween-20 in TBS for 15 min. The Alexa680 antimouse (#A10038) and IRDye 800CW antirabbit (#926-32213) secondary antibodies were from Thermo Fischer Scientific and LI-COR Biosciences, respectively. The membranes were scanned using an Odyssey Scanner and the blots were quantified using ImageStudio Lite v5.2.5 (LI-COR Biosciences).

### Deubiquitination assay

HEK293T cells were seeded in 10-cm cell culture dishes (Sarstedt AG) and then transfected with the indicated plasmids. After starvation, cells were lysed in a buffer containing 1% SDS, 50 mM Tris, 150 mM NaCl, 2 mM EDTA, pH 8.0, supplemented with Halt protease and phosphatase inhibitor cocktail (Thermo Scientific). The cell samples were passed through a 21-gauge needle to shear the DNA, sonicated and heated at 95 °C for 10 min, followed by a tenfold dilution in a buffer containing 50 mM Tris, pH 8.0, 150 mM NaCl, 2 mM EDTA, 1% NP-40, supplemented with Halt protease and phosphatase inhibitor cocktail (Thermo Scientific). After centrifugation for 5 min at 2500 rpm, immunoprecipitation of PDGFRβ and elution of proteins were performed, as described above.

### Immunofluorescence and image analysis

BJhTERT USP4 tet-inducible or CRISPR-Cas9 USP4-knockout cells were seeded on 10 × 10 mm coverslips, induced or not with doxycycline for 48 h, starved overnight in DMEM, supplemented with 0.1% FBS and stimulated with 20 ng/ml of PDGF-BB for indicated times. Cells were fixed in 3.7% of paraformaldehyde–PBS solution for 15 min at room temperature, permeabilized in 0.1% SDS, 1% BSA–PBS solution for 15 min, blocked in 1% BSA–PBS for 1 h and incubated with primary antibody overnight. Coverslips were washed 5 times, incubated with the secondary antibody for 50 min, washed 5 times, incubated with 1 µg/ml DAPI solution, mounted in Vectashield mounting media (Vector Labs, CA) and analysed by confocal microscopy. Images were acquired using ZEISS LSM700 inverted confocal microscope with numerical aperture 1.4 oil objective at Biological Visualization Facility (SciLife Lab, Uppsala University, Sweden) at 128 × 1128 pixels using Zen black software and high resolution AxioCam microscope camera. For presentation in the manuscript, images were exported as merged tiff files with 8xbit resolution, individual colour channels were adjusted for brightness equally on all images within the experiment. For image analysis of co-localization, original ZEN images were uploaded into Cell Profiler Imaging software [7] and automatic pipeline was created at SciLife BioImage Informatics Facility, Uppsala, Sweden, to estimate correlation between distribution of signals for individual channels across the image. Correlation was calculated using Pearson correlation coefficient with > 0 value for a possibility of correlation reaching to 1 when correlation is perfect; cutoff was placed at 0.2.

### RNA analysis

Tet-inducible BJhTERT cells were seeded at 0.5 × 10^5^ cell/dish in 10-cm Petri dishes, treated with 100 ng/ml of doxycycline for 8 h the next day, and maintained in DMEM, supplemented with 0.1% FBS with or without 100 ng/ml doxycycline with or without stimulation with 10 ng/ml PDGF-BB for 72 h. RNA was extracted with RNA extraction kit (Macherey–Nagel), 1 µg of total RNA was used for reverse transcription with High Capacity cDNA kit (Applied Biosystems), followed by quantitative PCR (qPCR) with SYBR green qPCR ready mix (Techtum) in CFX Opus 96 real time PCR system (BioRad). The following primers were used for qPCR: *HPRT* forward 5’cctggcgtcgtgattagtgat and reversed 5ʹ agacgttcagtcctgtccataa; *myc* forward 5ʹ aggctcctggcaaaaggtca and reversed 5ʹ ctgcgtagttgtgctgatgtg; *p21* forward 5ʹ-gtgtgagcagctgccgaagtca and reversed 5ʹ-tgacatggcgcctcctctgagt; *USP17* forward 5ʹ-tggatgatgccgaggtcacc and reversed 5ʹtgtctgtgtcttctgcgcca; *USP4* forward 5ʹ-actatgtattggtccctaccga and reversed 5ʹ-gcagtgcttgacaaacaggc.

### STAT3 transcription factor colorimetric assay

USP4- and USP17L22-tet-inducible BJhTERT cells were seeded at 0.5 × 10^5^ cells per 10 cm Petri dishes, induced or not with 100 ng/ml of doxycycline for 36 h, starved overnight in DMEM containing 0.1% FBS and 100 mg/ml doxycycline for the appropriate samples and stimulated with 20 ng/ml PDGF-BB for 15, 30 and 60 min. Binding of STAT3 to its interaction DNA element 5ʹ-TTCCCGGAA-3ʹ was analysed using a STAT3 transcription factor assay kit (Abcam, #ab207229) according to the manufacturer’s instructions. Nuclear extracts were isolated by swelling cells in hypotonic buffer (20 mM Hepes, pH = 7.5, 0.1 mM EDTA, 5 mM NaF, 10 µM Na_2_MoO_4_) with further addition of 0.5% NP-40 followed by a brief centrifugation at 4 °C for 30 s. Nuclear extracts were incubated with the microplate coated with the oligonucleotide sequence, washed, incubated with the primary STAT3 antibody, followed by antirabbit HRP-conjugated antibody, incubated with developing solution and absorbance was read on a spectrophotometer at OD 450 nm.

### Proliferation assay

Tet-inducible BJhTERT cells were seeded at 5000 cells/well in a 48-well plate (Sarstedt AG). After overnight cell starvation in DMEM, supplemented with 0.1% FBS, the cells were incubated in media containing 10% or 0.1% FBS, and/or the indicated PDGF-BB concentrations for 72 h. The cells were then washed with PBS to remove residual DMEM media and the CYQUANT Cell proliferation assay kit (#C7026, Thermo Scientific) was used according to manufacturer's specifications. The fluorescence of DNA-binding dye was measured using the Enspire multimode plate reader from Perkin Elmer set up with the excitation and emission filters at 480 nm and 520 nm, respectively.

### Statistical analysis

Statistical analyses were carried out using Microsoft Excel or GraphPad Prism version 7.0. The statistical significance of differences among mean values was determined by two-tailed t test with unequal variance; one-way ANOVA test was used for proliferation assay. *p < 0.05; **p < 0.01; ***p < 0.001. All experiments were repeated from three to five times.

## Results

### USP17L22 and USP4 deubiquitinate PDGFRβ

**Fig. 1:**
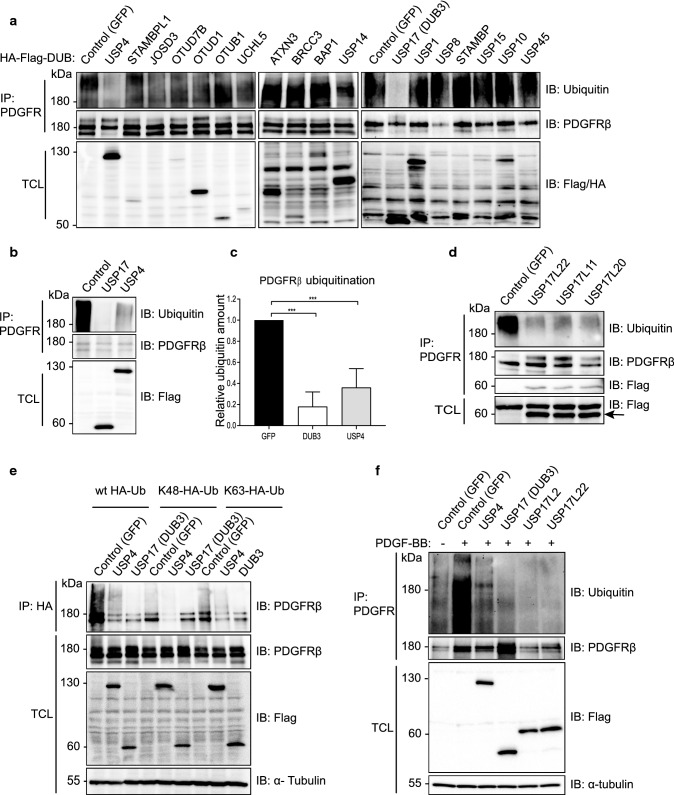
USP4 and USP17 are the deubiquitinases of PDGFRβ. **a** A DUB cDNA screen identifies USP17 and USP4 as deubiquitinating enzymes of PDGFRβ. Wild-type PDGFRβ and individual Flag- and HA-tagged DUB constructs were overexpressed in HEK293T, cells were serum-starved and stimulated with PDGF-BB (20 ng/ml) for 10 min, PDGFRβ was immunoprecipitated with anti-PDGFRβ antibody and eluates were immunoblotted for ubiquitin or PDGFRβ. Expression of DUBs in total cell lysates was confirmed by immunoblotting with anti-Flag (first two bottom panels) or HA (third bottom panel) antibodies. **b** USP4 and USP17 (DUB3) were selected and validated for the ability to remove ubiquitination from PDGFRβ. PDGFRβ was immunoprecipitated as described in panel **a** and eluates were immunoblotted for ubiquitin or PDGFRβ. Expression of DUBs in total cell lysates was confirmed with Flag antibody. **c** Quantification of PDGFRβ ubiquitination of at least three independent experiments as performed in panel **b** is shown; ***p < 0.001. **d** USP17L22, USP17L11 and USP17L20 interact with and deubiquitinate PDGFRβ. PDGFRβ and Flag-tagged USP17 isoforms were co-overexpressed in HEK293T; cells were then serum-starved and stimulated with PDGF-BB (20 ng/ml) for 10 min. PDGFRβ was immunoprecipitated and eluates were probed with a ubiquitin, PDGFRβ and Flag antibody. Expression of USP17 isoforms in total cell lysates is shown in the bottom panel. **e** USP4 and truncated USP17 (DUB3) remove Lys48- and Lys63-linked polyubiquitin chains from PDGFRβ. HEK293T cells were co-transfected with plasmids encoding Flag-GFP, Flag-USP4, Flag-DUB3, PDGFRβ, HA-ubiquitin or mutant HA-Lys48-Ub and HA-Lys63-Ub. Immunoprecipitation was performed with HA antibody and eluates were immunoblotted for PDGFRβ. The expression levels of PDGFRβ, Flag and α-tubulin in total cell lysates were also determined by immunoblotting. **f** USP4, USP17 (DUB3), full-length USP17L2 and USP17L22 isoforms remove ubiquitination from PDGFRβ under denaturing conditions. Lysates of cells that were co-expressing PDGFRβ with each of the indicated plasmids were boiled before immunoprecipitation with PDGFRβ and eluates were immunoprecipitated with HA antibodies and immunoblotted for ubiquitin and PDGFRβ. The expression levels of Flag and α-tubulin in total cell lysates were also determined by immunoblotting. *IP* immunoprecipitation, *IB* immunoblotting, *TCL* total cell lysates, *kDa* molecular mass in kilodalton

In order to identify deubiquitinating enzymes that may regulate ubiquitination and function of PDGFRβ, we performed an overexpression screen using a library of 64 Flag-HA-tagged DUBs [50]. For this purpose, individual DUBs were co-expressed with PDGFRβ in 293 T cell lines and their ubiquitination state was assessed after stimulation with PDGF-BB for 5 min by immunoprecipitation of PDGFRβ and immunoblotting for ubiquitin. Figure 1a shows selected DUBs and Supplementary Table 1 presents the summary of the screen. USP17 (named DUB3 in the library description) and USP4 efficiently deubiquitinated PDGFRβ, and were further analysed by quantification of their effects on deubiquitination of PDGFRβ (Fig. 1b,c; direct interaction between PDGFRβ and USP4 (Figure S1a) and USP17 (DUB3) was also detected (Figure S1b). It was noted that USP17 was extremely effective in removing ubiquitination from PDGFRβ, while USP4 mediated a partial effect when expressed at a similar level. The family of USP17 deubiquitinating enzymes consists of 30 highly similar proteins expressed from multiple genes on chromosomes 4 and 8 [5, 6] that share up to 90% sequence identity on the protein level and up to 96% identity on the DNA level. The expressed USP17 protein had a size of approximately 55 kDa instead of the expected 69 kDa, When the USP17 plasmid from the library was sequenced, it matched to the USP17L9p isoform and was found to contain a stop codon, which may represent a cloning artefact, leading to the expression of a truncated protein DUB3, which was nevertheless effective in removing ubiquitin chains as shown in Fig. 1 as well as interacting with PDGFRβ (Figure S1b). The expressed USP17 sequence aligned most closely to the USP17L22 family member, while missing RNA-binding and hyaluronan binding domains. We obtained a full-length Flag-tagged USP17L22 plasmid (GeneScript) in order to analyse the full-length protein. In addition, we mutated K488N and Q310T to create the USP17L11 isoform and K488N and Q310P to create the USP17L20 isoform and confirmed that all 3 full length USP17L proteins were equally effective in removing ubiquitination from PDGFRβ as the truncated USP17 (DUB3) (Fig. 1d). Moreover, by co-immunoprecipitation we detected an interaction between overexpressed full-length USP17 proteins and PDGFRβ in 293 T cells (Fig. 1d, third panel from the top). We also analysed USP17L2, which is the USP17 family member that is most dissimilar to USP17L22 and found that it too efficiently removed ubiquitination from PDGFRβ (Fig. 1f. Thus, it is likely that all USP17 family members are able to deubiquitinate PDGFRβ; we have used USP17L22 in further experiments.

Co-transfection of DUBs and PDGFRβ with mutant HA-tagged ubiquitin constructs that were able to form only K48- or only K63-linked chains, followed by immunoprecipitation of HA-ubiquitin and immunoblotting for PDGFRβ, revealed that both USP17 (DUB3) and USP4 were able to remove both K48- and K63-linked polyubiquitin chains from PDGFRβ (Fig. 1e). In order to exclude the possibility that the observed changes in deubiquitination of PDGFRβ were caused by ubiquitination status of other proteins that might have been bound to PDGFRβ during immunoprecipitation, we subjected samples to deubiquitination assay using denaturing conditions, where lysates were boiled before immunoprecipitation in order to remove all PDGFRβ-bound proteins. We were able to confirm that full length USP17L22 and USP17L2 efficiently removed ubiquitin marks from activated overexpressed PDGFRβ, while USP4 mediated a partial effect (Fig. 1f).

### USP17L22 and USP4 do not affect long-term stability of PDGFRβ

**Fig. 2:**
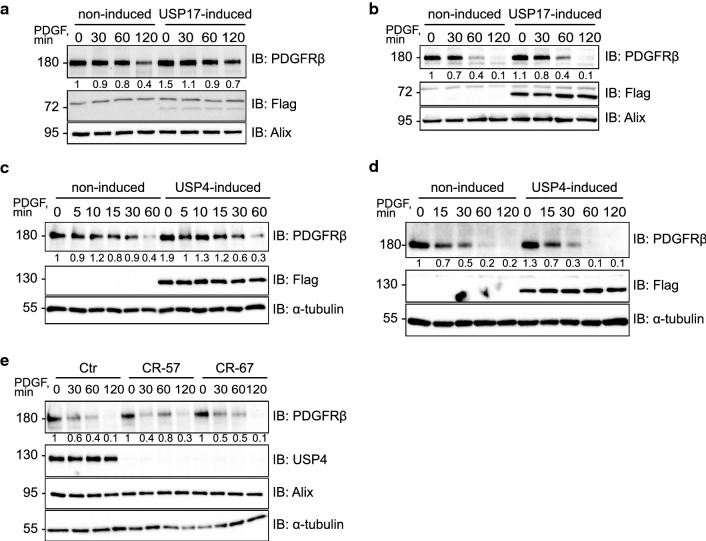
USP4 and USP17 do not stabilize the levels of PDGFRβ. **a**, **b** Induction of USP17L22 does not stabilize PDGFRβ levels. BjhTERT-USP17L22 tet-inducible cell line (**a**) and U2OS-USP17L22 tet-inducible cell line (**b**) were induced to express USP17L22 with doxycycline and pretreated with cycloheximide to block protein synthesis before stimulation with PDGF-BB (20 ng/ml) for indicated time periods. Expression levels of PDGFRβ, Flag-tagged USP4 and Alix as a loading control were determined by immunoblotting of total cell lysates. A representative experiment of three independent repeats is shown; expression levels of PDGFRβ relative to the loading control were quantified and the level of PDGFRβ in uninduced and unstimulated cells was set as 1. **c**, **d** Induction of USP4 does not stabilize PDGFRβ levels. BjhTERT-USP4 tet-inducible cell line (**c**) or U2OS-Usp4 tet-inducible cell line (**d**) was induced to express USP4 with doxycycline and pre-treated with cycloheximide before stimulation with PDGF-BB for indicated time periods. Expression levels of PDGFRβ, Flag and α-tubulin were determined in total cell lysates. A representative experiment of three independent repeats is shown; expression levels of PDGFRβ relative to the loading control were quantified and the level of PDGFRβ in uninduced and unstimulated cells was set as 1. **e** PDGFRβ degradation rate is not changed in BJhTERT-CRISPR-Cas9-USP4 knock-out fibroblasts. Wild-type BJhTERT and BJhTERT-CRISPR-Cas9-USP4 knockout cell line CR57 (sqRNA construct 57) and BJhTERT-CRISP-USP4 cell line CR67 (sqRNA construct 67) were pretreated with cycloheximide and stimulated with PDGF-BB (20 ng/ml) for the indicated time periods. Expression levels of PDGFRβ, USP4, Alix and α-tubulin were determined by immunoblotting of total cell lysates. Quantification is presented as shown in panels **a**–**d**. Molecular mass of molecular markers is indicated in kDa. *IB* immunoblotting, *TCL* total cell lysate

In order to assess the effect of USP4 and USP17 on PDGFRβ stability, we transiently overexpressed USP4 and USP17L22 in U2OS osteosarcoma cells that naturally express PDGFRβ. However, no appreciable effects on the stability of the receptor were observed (data not shown). In order to exclude the possibility of inefficient transfection in U2OS cells, we created stable tet-inducible cell lines, in which individual DUBs were induced in all cells in response to the addition of the tetracycline-derivative doxycycline to the media. Likewise, in these cells there was no significant effect on the PDGF-BB-induced rate of degradation of PDGFRβ, after USP17L22 had been induced for 48 h with doxycycline in BJhTERT (Fig. 2a) or U2OS (Fig. 2b) cells. Similarly, when USP4 was induced in tet-inducible BJhTERT (Fig. 2c) or tet-inducible U2OS (Fig. 2d) cells, no effect on ligand-induced degradation of PDGFRβ was observed.

In order to estimate how the loss of USP4 might affect the function of PDGFRβ, we created CRISPR-Cas9 mediated USP4 knockout BJhTERT cell lines. Consistently, in two independent mutant cell lines, that were created using G57 and G67 sgRNA (as described in the methods) and abbreviated CR-57 and CR-67, no effect on ligand-induced degradation of PDGFRβ was observed (Fig. 2e). It has been previously reported that USP17 is required for G1-S cell cycle progression [38] and serves as a deubiquitinating enzyme for a number of proteins crucial for cell viability, such as Cdc25a, and deletion of USP17 has been found to be lethal to the cells [50]. In agreement with this, we found that transient knockdown of USP17 with siRNA was toxic to the cells and lead to dramatic decrease of cell viability (as shown below in Fig. 6h), therefore it was not possible to create a stable USP17 CRISPR-Cas9 knockout cell line. This suggests that USP17 functions on multiple cellular substrates, maintaining cell viability in addition to deubiquitinating PDGFRβ.

### USP17L22 and USP4 act at different subcellular locations

**Fig. 3:**
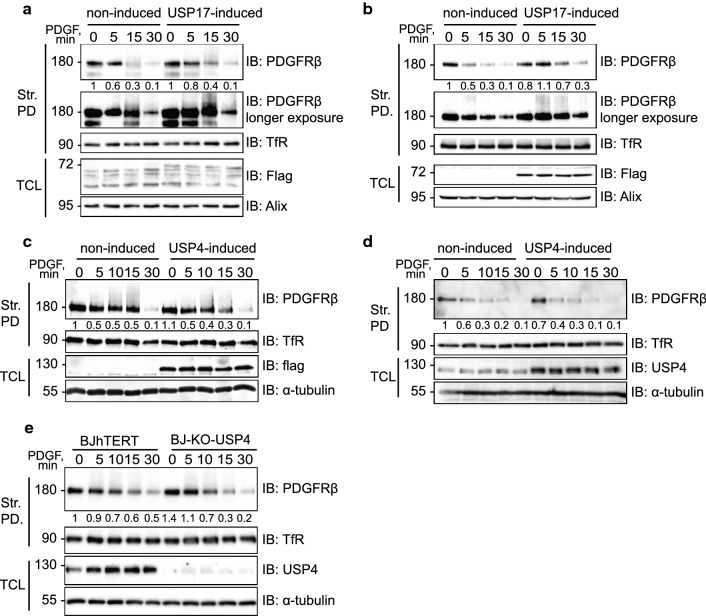
Internalization of PDGFRβ from the cell surface is affected by USP17, but not by USP4. **a**, **b** The internalization of PDGFRβ is delayed by induction of USP17L22. BJhTERT-USP17L22 tet-inducible cell line (**a**) or U2OS-USP17L22 tet-inducible cell line (**b**) was induced with doxycycline to express USP17L22, serum-starved and stimulated with PDGF-BB for the indicated periods of time. Biotinylated cell surface proteins were precipitated with streptavidin agarose and eluates were immunoblotted for PDGFRβ and transferrin receptor. The expression levels of Flag-tagged USP17L22 and Alix as a loading control in total cell lysates were also determined by immunoblotting. A representative experiment of three independent repeats is shown; expression levels of the cell surface PDGFRβ relative to the loading control were quantified using the lighter exposed blot and the level of PDGFRβ in uninduced and unstimulated cells was set as 1. **c**, **d** Induction of USP4 does not affect internalization of PDGFRβ. BJhTERT-USP4 tet-inducible cell line (**c**) or U2OS-USP4 tet-inducible cell line (**d**) was induced with doxycycline to express Usp4 and experiment was performed as described in panels **a** and **b**. Pulldown eluates were immunoblotted for PDGFRβ or transferrin receptor. The expression levels of Flag-tagged USP4 and Alix in total cell lysates were also determined by immunoblotting. The levels of PDGFRβ were quantified as described for panels **a** and **b**. **e** USP4 knockout does not affect the internalization rate of PDGFRβ. BJhTERT wildtype and USP4-CRISPR-Cas9 KO cells (BJ-KO-USP4) were stimulated with PDGF-BB (20 ng/ml) for the indicated time periods and treated as described in panel **a**. Biotinylated proteins were precipitated with streptavidin-agarose, immunoblotted for PDGFRβ and TfR. *Str. PD* streptavidin pulldown, *TfR* transferrin receptor, *IB* immunoblotting, *TCL* total cell lysate. The molecular mass of proteins is indicated in kilodalton

**Fig. 4:**
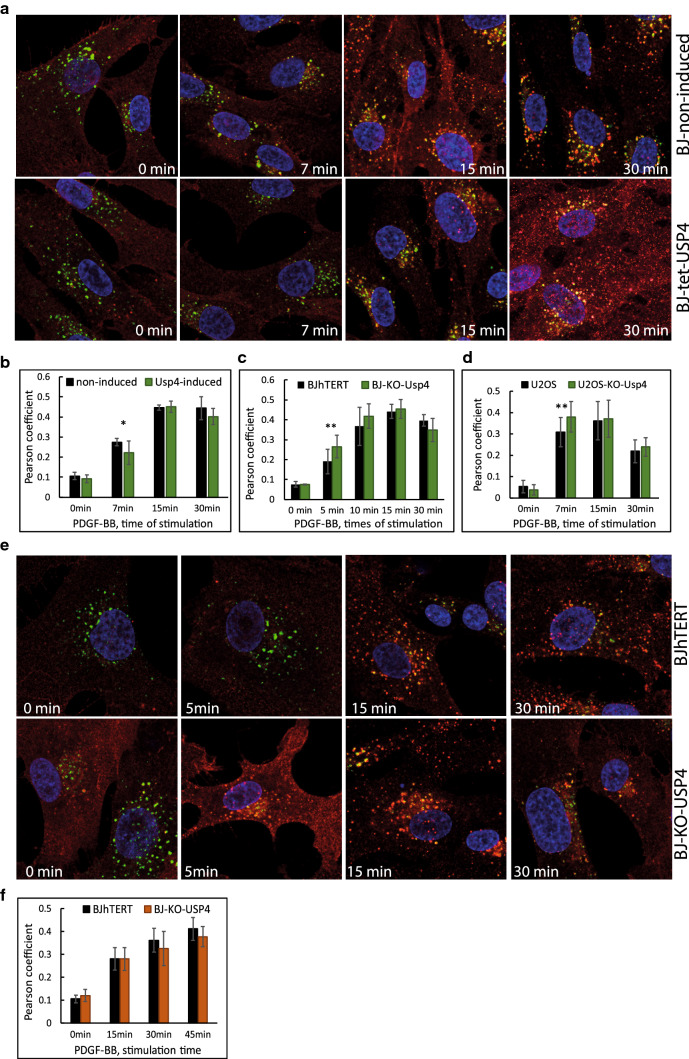
USP4 regulates the speed of co-localization of PDGFRβ with early endosomes. **a**, **b** Induction of USP4 delays co-localization of PDGFRβ with early endosomes. USP4 expression was induced in BJhTERT-USP4 tet-inducible cell line versus noninduced control cells. Representative images of co-localization (in yellow) of PDGFRβ (red) with early endosomal marker EEA1 (green) at indicated times of stimulation with PDGF-BB (**a**). Co-localization of PDGFRβ and EEA1 was quantified in 3 independent experiments and is presented as Pearson coefficient of correlation of distribution of signals for each analysed channel in 15–35 cells per condition. *p < 0.05 (**b**). **c**–**e** CRISPR-Cas9-mediated knockout of USP4 leads to faster co-localization of PDGFRβ with early endosomes. Co-localization of PDGFRβ and EEA1 marker was quantified and presented as in panel **b** for BJ-CRISPR-Cas9 knockout cells versus BJhTERT control cells (**c**) and U2OS CRISP-USP4 knockout cells versus U2OS control cells (**d**). **e** Representative images of co-localization (in yellow) of PDGFRβ (red) with early endosomal marker (green) at indicated times of stimulation with PDGF-BB of the experiments quantified in panel **c** are presented. **f** Co-localization between PDGFRβ and late endosomal marker (Rab7) is presented for BJhTERT CRISP-USP4 knockout cells versus BJhTERT control cells

The lack of long-term stabilization of endogenous PDGFRβ in response to the induction of USP4 and USP17 suggested that deubiquitination of PDGFRβ by these DUBs did not extend the half-life of PDGFRβ. In order to explore the possibility that USP4 and USP17 may regulate subcellular trafficking of PDGFRβ in response to PDGF-BB stimulation, we analysed the effect of USP4 and USP17 on internalization of PDGFRβ from the cell surface. We found that the induction of USP17 led to a slightly increased presence of PDGFRβ on the cell surface in USP17L22-tet-inducible BJhTERT cells, despite low induction of USP17L22 cells **(**Fig. 3a) and more clearly in USP17L22-tet-inducible U2OS cells where the induction was stronger (Fig. 3b), while no effect on PDGFRβ internalization was seen upon induction of USP4 in both tet-inducible BJhTERT (Fig. 3c) and in U2OS (Fig. 3d) cells. Consistently, the amount of cell surface-localized PDGFRβ was not significantly different between PDGF-BB stimulated normal BJhTERT and USP4-CRISPR-Cas9 knockout fibroblasts (BJ-KO-USP4) after stimulation with PDGF-BB (Fig. 3e). Although we sometimes observed fluctuations of total levels of PDGFRβ in unstimulated cells both on the cell surface and in the total cell extracts, these differences were not significant. Thus, our results suggest that USP17 prolongs the presence of PDGFRβ on the cell membrane by affecting the internalization of the receptor from the cell surface, while USP4 may deubiquitinate PDGFRβ at other cellular locations. We explored the role of USP4 in the downstream sorting events and found that the co-localization of PDGFRβ with early endosomes upon induction of USP4 in tet-inducible BJhTERT fibroblasts, was decreased at 7 min of stimulation with PDGF-BB, as compared to the control cells (Fig. 4a, b). The staining pattern of early endosomes was unchanged, while the differences in co-localization with early endosomes appeared to be associated with different timing of PDGFRβ clustering (Figure S2) and subsequent delivery to the early endosomes. Thus, the presence or absence of ubiquitin marks may regulate the sorting of PDGFRβ via clathrin-coated pits. Consistently, upon depletion of USP4 in BJ-KO-USP4 cells (Fig. 4c, e) and in U2OS-USP4 knockout cells (U2OS-KO-USP4) (Fig. 4d), there was significant co-localization of clustered PDGFRβ with the early endosomal marker EEA1 while co-localization of PDGFRβ with the late endosomal marker Rab7 was not significantly affected (Fig. 4f).

### Deubiquitination of PDGFRβ regulates the timing of activation of STAT3

**Fig. 5:**
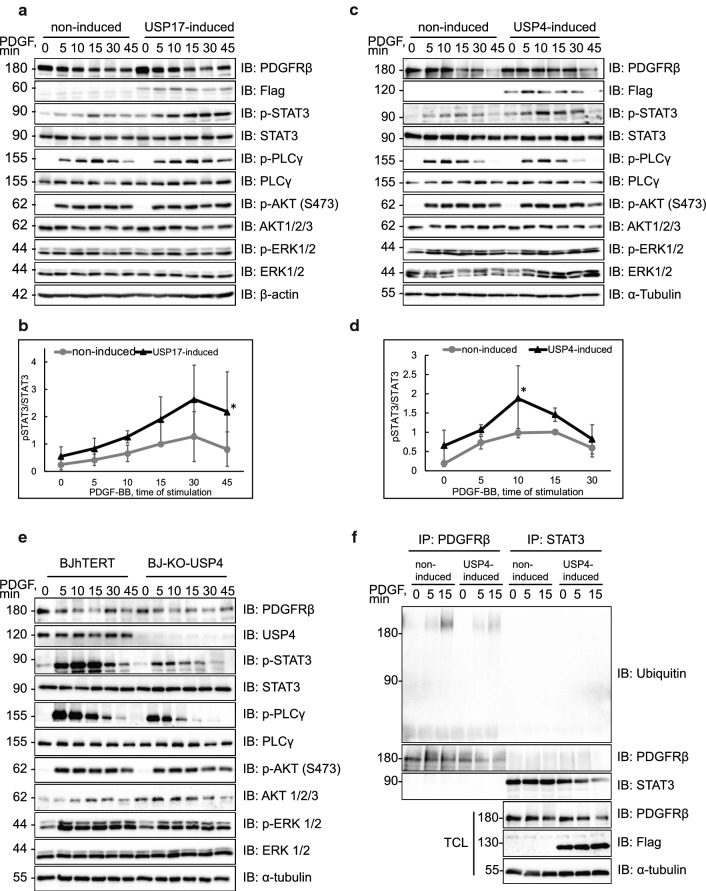
USP4 and USP17L22 affect the timing of activation of STAT3. **a**, **b** Induction of USP17L22 results in a shift of STAT3 activation to later time points in BJhTERT inducible cells. Expression of Flag-tagged USP17L22 was induced with doxycycline, cells were serum-starved and stimulated with PDGF-BB (20 ng/ml) for the indicated time periods. Expression of total and phosphorylated proteins was determined in total cell lysates using antibodies against PDGFRβ, Flag, STAT3 (pY705), STAT3, PLCγ (pY783), PLCγ, pS473 Akt1/2/3, Akt1/2/3, pThr202/pThr204 Erk1/2, Erk1/2 and β-actin (**a**). Phosphorylated STAT3 (pSTAT3) versus total STAT3 levels were quantified in three experiments; peak of phosphorylation at 10 min of stimulation with PDGF-BB was set as 1. Standard deviation is shown between repeats; *p < 0.05 (**b**). **c**, **d** Induction of USP4 increases the STAT3 activation in BJhTERT-USP4 inducible cells. BJhTERT-USP4 inducible cells were treated as described and immunoblotting was performed for proteins as in panel **a**; α-tubulin was used as loading control. Activated pSTAT3 relative to STAT3 protein levels were quantified in three independent experiments as in panel **b**; *p < 0.05 (**d**). **e** CRISPR-Cas9 knockout of USP4 decreases the STAT3 activation in BJhTERT-CRISPR-Cas9-USP4 knockout cells. The control BJhTERT and CRISPR-Cas9-USP4 knockout BJhTERT cells were starved overnight and stimulated with PDGF-BB (20 ng/ml) for the indicated time periods. Immunoblotting was performed for the indicated proteins, as in panels **a**–**d**. **f** USP4 does not deubiquitinate STAT3. After USP4 induction and serum starvation, BJhTERT-USP4 cells were stimulated with PDGF-BB (20 ng/ml) for the indicated time periods, lysates were divided and immunoprecipitated with anti-PDGFRβ (CTβ) or anti-STAT3 antibodies. Eluates were immunoblotted for ubiquitin. Levels of PDGFRβ, Flag-USP4 and α-tubulin were determined by immunoblotting (IB)

We next investigated the impact of deubiquitinating activity towards PDGFRβ on the subsequent signalling events. Interestingly, we found that both USP4 and USP17 mainly affected the PDGFRβ-mediated activation of STAT3, as detected by phosphorylation of Tyr705, which is a marker for its activation [32], while the activation of certain other signalling effectors (such as PLCγ) was not significantly affected. Induction of USP17L22 in BJhTERT fibroblasts enhanced the activation of STAT3 **(**Fig. 5a, c), which was statistically significant at 45 min, while USP4 induction produced a little increase and slight shift in the peak of STAT3 activation, which was significant at 10 min of stimulation with PDGF-BB (Fig. 5b, d). Conversely, deletion of USP4 in BJhTERT-CRISPR-Cas9 knockout fibroblasts led to decreased activation of STAT3 in response to PDGF-BB (Fig. 5e). These findings are in agreement with a previous report, showing that internalization and intracellular trafficking of PDGFRβ is necessary for efficient activation of STAT3 and that STAT3 is activated both at the plasma membrane and at the early endosomes [25]. We could not detect any ubiquitination of the STAT3 protein itself (Fig. 5f and S3) or any effect of overexpression of USP4 and USP17 on the total levels of STAT3, thus ruling out the possibility that these DUBs directly regulate STAT3. It is therefore likely that USP4 and USP17 affect the activation of STAT3 by acting on PDGFRβ at both of these cellular locations; USP17 by retaining PDGFRβ longer at the plasma membrane and USP4 by regulating the timing of early delivery of PDGFRβ to early endosomes.

### STAT3 transcriptional activity and expression of STAT3 target genes is affected by induction of USP17 and USP4

**Fig. 6:**
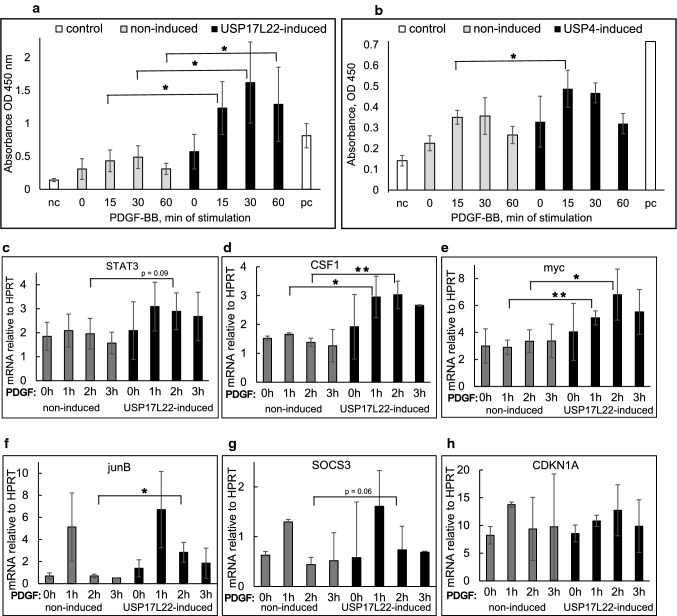
Activation of STAT3 promotes increased DNA binding and results in acute induction of STAT3 target genes. **a**, **b** Transcriptional activity of STAT3 is increased upon induction of DUBs and PDGF-BB stimulation. USP17L22 (**a**) or USP4 (**b**) were induced in the BJhTERT tet-inducible cell lines, cells were starved overnight and stimulated with PDGF-BB for 0, 15, 30 and 60 min. 20 µg of nuclear extracts were incubated with STAT3 binding element 5ʹ-TTCCCGGAA-3ʹ in a microplate and binding was quantified by colorimetric ELISA. Positive control was 5 µg of HepG2 cells stimulated with 100 ng/ml IL-6 (as supplied in the kit). The value for negative control was obtained by incubating each of the unstimulated or stimulated for 15, 30 or 60 min nuclear extracts from the uninduced BJhTERT cells with the addition of 20 pmol of unbound oligonucleotide that competed with the binding of activated STAT3 to the plate (as supplied in the kit). Absorbance values were plotted for three independent repeats; standard deviation is shown. The statistical significance was determined by one-tailed t test with unequal variance; *p < 0.05. **c**–**h** Induction of USP17L22 promotes acute upregulation of STAT3 target genes. USP17L22 was induced in the BJhTERT tet-inducible cell line which were stimulated with PDGF-BB for 1, 2 or 3 h and the expression of mRNA for *STAT3* (**c)**, *CSF-1* (**d**), *myc* (**e**), *SOCS3* (**f**), *junB* (**g**) and *CDKN1A* (**h**) was determined by quantitative PCR

We proceeded to verify if changes in the phosphorylation status of STAT3 induced by overexpression of the DUBs during PDGF-BB stimulation lead to differences in transcriptional activity of activated STAT3. To this end we quantified the binding of activated STAT3 to its consensus binding element immobilized onto a microplate by an ELISA type assay upon induction of DUBs in BJhTERT tet-inducible cell lines and PDGF-BB stimulation for 15, 30 and 60 min. We observed a dramatic and significant PDGF-BB-inducible increase in the STAT3 binding when USP17 was induced in the cells (Fig. 6a), while induction of USP4 lead to a weak increase in the binding of activated STAT3 to its consensus binding element which was significant at 15 min of stimulation with PDGF-BB (Fig. 6b). We further analysed the effect of induction of DUBs and PDGF-BB stimulation on the short-term transcriptional changes for some STAT3 target genes that could be relevant in PDGF-BB signalling. We indeed observed that strong binding of STAT3 to its binding element upon induction of USP17 as detected by ELISA correlated to the increased gene expression of *STAT3* itself (Fig. 6c), colony-stimulating factor-1 (*CSF1*) (Fig. 6d) and *myc* (Fig. 6e), while leading to somewhat increased and prolonged induction of *junB* (Fig. 6f), insignificant increase of suppressor of cytokine signalling 3 (*SOCS3*) (Fig. 6g) and no effect on the expression of *CDKN1A* (Fig. 6h).

**Fig. 7:**
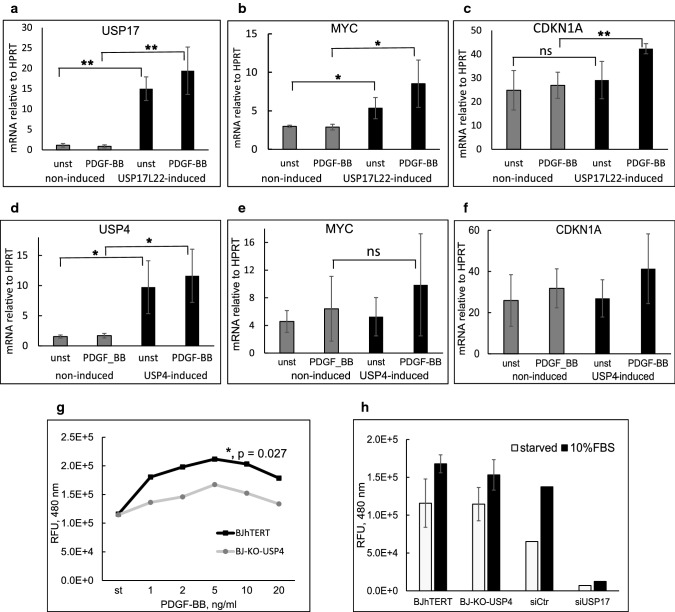
Induction of USP17 and USP4 upregulate gene expression of *myc* and *CDKN1A* affecting cell proliferation. **a**–**c** USP17L22 promotes upregulation of *myc* and *CDKN1A* transcription in response to PDGF-BB. USP17L22 was induced in the BJhTERT tet-inducible cell line (**a**) and the expression of mRNA for *myc* (**b**) and *CDKN1A* (**c**) was determined by quantitative PCR. **d**–**f** USP4 affects expression of *myc* but not *CDNK1A* in response to PDGF-BB. USP4 was induced in BJhTERT tet-inducible cell line (**d**) and mRNA for *myc* (**e**) and *CDKN1A* (**f**) was determined by quantitative PCR. **g** Deletion of USP4 in BJhTERT CRISPR-Cas9 knockout cells decreases proliferative response to PDGF-BB, as compared to BJhTERT control cells. Fibroblasts were cultured for 3 days in the absence or the presence of increasing concentrations of PDGF-BB, cell proliferation was determined by measurement of DNA-intercalating fluorescent dye using CYQUANT proliferation assay. Relative fluorescent counts relate to the number of cells in the samples. **h** Total cell growth was not affected by deletion of USP4 in BJhTERT CRISPR-Cas9 knockout cells, while transient knockdown of USP17 in BJhTERT led to cell death. CYQUANT proliferation assay was used to measure relative number of cells growing under conditions of starvation in 1% FBS versus complete media for BJhTERT fibroblasts, BJ-CRISP-Cas9-USP4 fibroblasts (BJ-KO-Usp4) and BJhTERT fibroblasts transiently transfected with nontargeting control siRNA or USP17L9P siRNA

In order to investigate the long-term consequences of aberrant STAT3 activation upon induction of USP4 and USP17, we analysed expression levels of STAT3 target genes, i.e. *myc* and *CDKN1a (p21),* that are known to have opposing functions in the regulation of cell proliferation [1]. Interestingly, we found that induction of USP17L22 (Fig. 7a) in BJhTERT cells during 3 days of treatment of cells with PDGF-BB led to significant upregulation of expression of both *myc* (Fig. 7b) and *p21* (Fig. 7c), as compared to noninduced PDGF-BB-treated cells. Similarly, upon induction of USP4 (Fig. 7d), mRNA expression levels of *myc* in response to PDGF-BB was elevated compared to the noninduced cells (Fig. 7e), while expression of *CDKN1A* was somewhat elevated (Fig. 7f). The effects of USP4 induction followed the same trend as the effects of USP17L22, however, due to lower and variable levels of induction no statistical significance between repeats could be obtained.

### PDGF-BB-induced cell proliferative response is dysregulated upon manipulation of USP17 and USP4 levels

In order to determine the functional significance of the PDGFRβ ubiquitination status, we analysed PDGF-BB-induced proliferation, contraction and migration of cells upon induction and deletion of USP17L22 or USP4 in BJhTERT cells. CRISPR-Cas9-mediated deletion of USP4 led to decreased proliferative response to PDGF-BB (Fig. 7g), while cell growth in full media (DMEM, supplemented with 10% FBS) was not affected (Fig. 7h). As mentioned above, USP17 has been demonstrated to act on several cell cycle proteins and indeed we observed that even transient USP17 depletion adversely affected viability of BJhTERT fibroblasts (Fig. 7h), which may be explained by the fact that USP17 acts on other substrates that are essential for cell cycle progression. When USP17L22 or USP4 were induced in tet-inducible cell lines, we did not observe any significant changes in proliferative response to PDGF-BB (data not shown). This may be explained by opposing effects of STAT3-mediated activation on both mitogenic (downstream of myc) and antiproliferative (downstream of CDKN1a (p21)) signalling pathways, leading to different effects on proliferation of different cell types. No consistent effects on PDGF-BB-induced cell contraction or cell migration were observed upon induction or deletion of USP4 or USP17 (data not shown).

## Discussion

Ligand-mediated dimerization and activation of RTKs, including PDGFRβ, promote activation of signalling pathways, but also initiates internalization and downregulation of receptors [20, 21] which is controlled by ubiquitination of the receptor [35]. The existence of more than 600 ubiquitin ligases in the human genome is indicative of the complexity of this type of posttranslational modification [24, 29] while deregulation of ubiquitination has been associated with the development of various diseases, including cancer [43]. It has become evident that deubiquitinating enzymes play an important role in balancing the action of ubiquitin ligases, defining the level and type of ubiquitination, which determines not only degradation rate but also speed of internalization, sorting mechanisms and signalling downstream of activated receptors. The importance of direct deubiquitination for internalization and sorting of RTKs has been shown for EGFR [2, 37], HER2 involving POH1 [34] and NGF receptor TRKA involving CYLD [14]. In this work we identified USP17 and USP4 as deubiquitinating enzymes that regulate trafficking and signalling of PDGFRβ.

In our study we have shown that both USP17 and USP4 directly deubiquitinate PDGFRβ; thus, the observed removal of PDGF-BB-induced ubiquitination was not due to deubiquitination of some PDGFRβ-bound proteins that co-immunoprecipitated with the receptor. However, the possibility that USP17 and USP4 act on E3 ligases, thereby decreasing addition of ubiquitin marks on PDGFRβ cannot be excluded. This possibility remains to be further explored. Interestingly, we have not seen any appreciable stabilization effect on the level of PDGFRβ after overexpression of USP17 or USP4 or by knockdown of USP4. Thus, it appears that the action of the two DUBs is not directed towards reversing the proteasomal or lysosomal degradation of PDGFRβ. However, we were able to monitor a transient delay of PDGFRβ trafficking during the short course of PDGF-BB stimulation that correlated with changes in activation of STAT3. This suggests that the subcellular localization of the DUBs plays an important role for the function of deubiquitinating enzymes on PDGFRβ and also confirms the previously reported findings that the process of PDGFRβ endocytosis defines the PDGF-BB-mediated activation of STAT3 [25]. We observed that induction of USP17L22 caused a delay in the internalization of PDGFRβ from the cell surface that is consistent with its reported general role in the regulation of endocytosis of EGFR [26]. Surprisingly, USP4 did not act in a similar fashion, in fact, deletion of USP4 speeded up the sorting of PDGFRβ towards early endosomes. Notably, USP8 has been reported to act on EGFR in a similar fashion; deletion of USP8 promotes faster internalization of EGFR, demonstrating that it regulates endosomal trafficking of EGFR [2]. In our study, deubiquitination of PDGFRβ by USP4 prolonged its time of signalling between the plasma membrane and early endosomes, while depletion of USP4 speeded up the sorting and decreased the amplitude of activation of STAT3.

The multifunctionality of DUBs and the variety of the substrates that they may work on represents a challenge in identification of the functional role that the DUBs may have on PDGFRβ signalling. Both overexpression and downregulation of USP4 have been associated with tumorigenesis [16, 33, 56], while USP17 has been reported to control proliferation, cell cycle, apoptosis and stemness in different systems. The USP17 family of DUBs has 30 members that are crucial for the progression of the cell cycle, but can also adversely regulate cell proliferation and apoptosis [13]. USP17 was previously shown to inhibit proliferation by inhibiting phosphorylation of the downstream kinases MEK1/2 and ERK1/2 MAP-kinase by blocking Ras membrane localization and activation [4]. Conversely, USP17 has been reported to promote mitogenic gene expression and cell proliferation by deubiquitinating transcription factor Elk-1 that is induced by the activation of ERK1/2 [12]. Remarkably, in our study manipulations of the levels of expression of either USP17 or USP4 during PDGF-induced signalling led to a consistent impact on the activation of STAT3, which also translated into an increased transcriptional activity of STAT3 and affected acute some STAT3 target genes. Thus, the above reported adverse effects of USP17 overexpression may be explained, at least in part, by the upregulation of two functionally conflicting STAT3 target genes, *myc* and *CDKN1A* in response to PDGF-BB stimulation; myc promotes proliferation and oncogenic transformation, while CDKN1A suppresses the progression of the cell cycle. USP4 had milder but similar effect on the expression of *myc*, but not on *CDKN1A*, which is consistent with weaker impact on the activation of STAT3. This demonstrates the importance of regulation of the timing of the subcellular trafficking of the receptor by DUBs, which leads to selective activation of certain signalling pathways even if the impact on PDGFRβ trafficking is minor. In our study, we observed that PDGF-induced cell proliferation was affected by the function of USP17 and USP4, while we could not detect any appreciable changes in migration and contraction of fibroblasts. However, since both DUBs promote the activation of STAT3, it is difficult to assess the individual impact of each DUB on PDGFRβ-induced proliferation. For example, the knockdown of USP4 may lead to compensation by USP17 or other DUBs that may affect PDGFRβ ubiquitination simultaneously. In addition, the ability of DUBs to work on multiple substrates contributes to the functional output.

In conclusion, we have found that USP4 and USP17 are deubiquitinating enzymes acting on the PDGFRβ capable of removing both Lys48- and Lys63-linked polyubiquitin chains. Although this did not affect ligand-induced PDGFRβ degradation, it did modulate its trafficking in response to PDGF-BB; USP17 slowed receptor clearance from the cell surface, whereas USP4 had a role in fine-tuning the kinetics of PDGFRβ arrival to the early endosome. The altered PDGFRβ trafficking correlated to altered STAT3 activation and the expression of its target genes, including *myc* and *p21* that are important in the control of cell proliferation.

### Supplementary Information

Below is the link to the electronic supplementary material.

Supplementary Figure S1. USP4 (a) and USP17 (DUB3) (b) interact with PDGFRβ. Flag-HA-tagged GFP or DUB constructs were overexpressed together with PDGFRβ in 293T cells, starved and stimulated with PDGF-BB for indicated times. Proteins were immunoprecipitated as indicated and eluates were blotted for PDGFRβ and Usp4 (a) or PDGFRβ and USP17 (DUB3) (b). The expression of PDGFRβ, flag-HA-tagged USP4 and flag-HA-tagged USP17 (DUB3) was confirmed in total cell lysates (TCL), α-tubulin was used as loading control. (EPS 4355 kb)

Supplementary figure S2. Single channels for co-localization images as presented in Figure 4a. (EPS 25549 kb)

Supplementary Figure S3. STAT3 protein is not ubiquitinated in USP17L22-inducible BJhTERT cell line. Co-immunoprecipitation and immunoblotting was performed for PDGFRβ and STAT3 in USP17L22-inducible cell line as described for USP4-inducible cell line in Figure 5f. The membrane from ubiquitination blot was cut and reblotted for STAT3. Expression of proteins is confirmed in total cell lysates (TCL). (EPS 3699 kb)

Supplementary file4 (DOCX 17 kb)

## Data Availability

All data, constructs and cell lines generated and analysed during this study are available from the corresponding author on reasonable request.
