# Supplementary material for: Deubiquitinating enzymes USP4 and USP17 finetune the trafficking of PDGFRβ and affect PDGF-BB-induced STAT3 signalling
Source: Cell Mol Life Sci. 2022 Jan 21;79(2):85. doi: 10.1007/s00018-022-04128-1 (PMC8782881; doi:10.1007/s00018-022-04128-1)
Supplement: Supplementary file 4 — Supplementary file4 (DOCX 17 kb) [file 18_2022_4128_MOESM4_ESM.docx]

| DUB | Size (kDa) | Deubiquitination of PDGFRβ is decreased | DUB expression in TCL/detected with antibody |  |  |
| --- | --- | --- | --- | --- | --- |
| ATXN3 | 41 | no | flag |  |  |
| [BAP1](http://www.addgene.org/22539/) | 80 | no | flag |  |  |
| [BRCC3](http://www.addgene.org/22540/) | 36 | no | flag |  |  |
| [COPS5](http://www.addgene.org/22541/) | 37 | no | nd |  |  |
| [COPS6](http://www.addgene.org/22542/) | 36 | no | flag |  |  |
| [CYLD](http://www.addgene.org/22544/) | 107 | no | HA |  |  |
| [DUB3](http://www.addgene.org/22593/) | 60 | yes | flag/HA |  |  |
| [EIF3S3](http://www.addgene.org/22545/) | 40 | no | nd |  |  |
| [EIF3S5](http://www.addgene.org/22546/) | 38 | no | flag |  |  |
| [Flag-HA-GFP control](http://www.addgene.org/22612/) | 27 | no | flag |  |  |
| [JOSD1](http://www.addgene.org/22547/) | 23 | no | nd |  |  |
| [JOSD2](http://www.addgene.org/22548/) | 21 | no | nd |  |  |
| [JOSD3](http://www.addgene.org/22549/) | 32 | no | flag |  |  |
| [OTUB1](http://www.addgene.org/22551/) | 31 | no | flag |  |  |
| [OTUB2](http://www.addgene.org/22552/) | 27 | no | flag |  |  |
| [OTUD1](http://www.addgene.org/22553/) | 51 | no | flag |  |  |
| [OTUD4](http://www.addgene.org/22594/) | 124 | no | flag |  |  |
| [OTUD5](http://www.addgene.org/22610/) | 60 | no | flag |  |  |
| [OTUD6B](http://www.addgene.org/22555/) | 34 | no | nd |  |  |
| [OTUD7B](http://www.addgene.org/22550/) | 92 | no | flag |  |  |
| [PARP11](http://www.addgene.org/22556/) | 40 | no | nd |  |  |
| [PSMD14](http://www.addgene.org/22557/) | 35 | no | flag |  |  |
| [PSMD7](http://www.addgene.org/22558/) | 37 | no | flag |  |  |
| [STAMBP](http://www.addgene.org/22560/) | 48 | no | HA |  |  |
| [STAMBPL1](http://www.addgene.org/22559/) | 50 | no | flag |  |  |
| [UCHL1](http://www.addgene.org/22563/) | 25 | no | nd |  |  |
| [UCHL3](http://www.addgene.org/22564/) | 26 | no | nd |  |  |
| [UCHL5](http://www.addgene.org/22565/) | 38 | no | flag |  |  |
| [USP1](http://www.addgene.org/22596/) | 88 | no | flag/HA |  |  |
| [USP10](http://www.addgene.org/22543/) | 87 | no | HA |  |  |
| [USP11](http://www.addgene.org/22566/) | 110 | no | flag |  |  |
| [USP13](http://www.addgene.org/22568/) | 97 | no | flag |  |  |
| [USP14](http://www.addgene.org/22569/) | 56 | no | flag |  |  |
| [USP15](http://www.addgene.org/22570/) | 112 | no | flag |  |  |
| [USP16](http://www.addgene.org/22595/) | 93 | no | nd |  |  |
| [USP18](http://www.addgene.org/22572/) | 43 | no | nd |  |  |
| [USP2](http://www.addgene.org/22577/) | 68 | no | flag |  |  |
| [USP20](http://www.addgene.org/22573/) | 102 | no | flag |  |  |
| [USP21](http://www.addgene.org/22574/) | 63 | no | flag |  |  |
| [USP22](http://www.addgene.org/22575/) | 60 | no | flag |  |  |
| [USP25](http://www.addgene.org/22597/) | 122 | no | flag |  |  |
| [USP26](http://www.addgene.org/22598/) | 104 | no | nd |  |  |
| [USP28](http://www.addgene.org/22576/) | 122 | no | flag |  |  |
| [USP29](http://www.addgene.org/22599/) | 104 | no | flag/HA |  |  |
| [USP3](http://www.addgene.org/22582/) | 59 | no | flag |  |  |
| [USP30](http://www.addgene.org/22578/) | 59 | no | flag |  |  |
| [USP33](http://www.addgene.org/22601/) | 107 | no | flag/HA |  |  |
| [USP36](http://www.addgene.org/22579/) | 123 | no | nd |  |  |
| [USP37](http://www.addgene.org/22602/) | 110 | no | flag/HA |  |  |
| [USP38](http://www.addgene.org/22580/) | 116 | no | flag |  |  |
| [USP39](http://www.addgene.org/22581/) | 65 | no | flag |  |  |
| USP4 | 109 | yes | flag/HA |  |  |
| [USP42](http://www.addgene.org/22603/) | 145 | no | nd |  |  |
| [USP43](http://www.addgene.org/22583/) | 123 | no | flag |  |  |
| [USP44](http://www.addgene.org/22604/) | 81 | no | flag/HA |  |  |
| [USP45](http://www.addgene.org/22605/) | 45 | no | flag/HA |  |  |
| [USP46](http://www.addgene.org/22584/) | 42 | no | nd |  |  |
| [USP48](http://www.addgene.org/22585/) | 119 | no | flag |  |  |
| [USP49](http://www.addgene.org/22586/) | 79 | no | nd |  |  |
| [USP5](http://www.addgene.org/22590/) | 96 | no | flag/HA |  |  |
| [USP50](http://www.addgene.org/22588/) | 39 | no | nd |  |  |
| [USP52](http://www.addgene.org/22589/) | 135 | no | nd |  |  |
| [USP53](http://www.addgene.org/22606/) | 120 | no | flag/HA |  |  |
| [USP7](http://www.addgene.org/22591/) | 128 | no | flag/HA |  |  |
| [USP8](http://www.addgene.org/22608/) | 127 | no | HA |  |  |
| [USPL1](http://www.addgene.org/22607/) | 120 | no | nd |  |  |
| [VCPIP1](http://www.addgene.org/22592/) | 134 | no | nd |  |  |
| [YOD1](http://www.addgene.org/22554/) | 38 | no | flag |  |  |
|  |  |  |  |  |  |
| **Supplementary Table S1**. Flag-HA-tagged DUB constructs from the library that were co-expressed with PDGFRβ. The effect on ubiquitination of PDGFRβ and expression of the DUBs in total cell lysates as detected by flag or HA antibodies is indicated; nd – not detected. | | | | |  |
|  |  |  |  |  |  |
|  |  |  |  |  |  |
|  |  |  |  |  |  |
